# Supplementary material for: Bone marrow adipose tissue is a unique adipose subtype with distinct roles in glucose homeostasis
Source: Nat Commun. 2020 Jun 18;11:3097. doi: 10.1038/s41467-020-16878-2 (PMC7303125; doi:10.1038/s41467-020-16878-2)
Supplement: Supplementary file 1 — Supplementary Information [file 41467_2020_16878_MOESM1_ESM.pdf]

SUPPLEMENTARY INFORMATION

Bone marrow adipose tissue is a unique adipose subtype with distinct roles in systemic glucose homeostasis

Suchacki et al.

Inventory of Supplementary Information:

Supplementary Table 1 – Table of key resources

Supplementary Figure 1, Related to Figure 1 – BMAT is transcriptionally distinct from white, brown and beige adipose tissues.

Supplementary Figure 2, Related to Figure 1 – BM adipocytes in humans are transcriptionally distinct from WAT adipocytes.

Supplementary Figure 3, Related to Figure 5 – Effects of cold exposure on energy homeostasis and gene expression in BAT, iWAT and bone.

Supplementary Figure 4, Related to Figure 5 – Effects of cold exposure on BM adiposity and adipocyte morphology in iWAT and bone.

Supplementary Figure 5, Related to Figure 5 – Effects of cold exposure on transcript expression in BAT, iWAT and bone.

Supplementary Figure 6, Related to Figure 7 – Human BMAT is functionally distinct from BAT.

Supplementary Table 1 – Table of key resources

REAGENT or RESOURCE	SOURCE	IDENTIFIER
BIOLOGICAL SAMPLES		
Human bone marrow (*cohort 1*)	Orthopedic and Traumatology Department, Ospedali Riuniti, Ancona, Italy	N/A
Human white adipose tissue (*cohort 1*)	Hepatobiliary and Abdominal Transplantation Surgery, Department of Experimental and Clinical Medicine, Università Politecnica delle Marche, Ancona, Italy	N/A
Human bone marrow and white adipose tissue (*cohort 2*)	Edinburgh Adipose Tissue Biobank	N/A
CHEMICALS		
Collagenase Type I	Worthington Biochemicals	LS004196
18F-Fluorodeoxyglucose	Edinburgh Clinical Research Imaging Centre (Edinburgh, UK)	N/A
Insulin	Eli Lilly (Indianapolis, USA)	Humulin S
Ribozol^{TM}	Amresco (USA)	N580
Osmium Tetroxide	Agar Scientific (UK)	AGR1022
OLIGONUCLEOTIDES		
See Table 2	This paper	N/A
EXPERIMENTAL MODELS: Organisms/Strains		
Mouse: C57BL/6J	Charles River	027
Rabbit: New Zealand White	Envigo	Hsdlf:NZW
SOFTWARE		
Analyze	AnalyzeDirect (Overland Park, KS, USA)	v12.0
Bioconductor	Bioconductor	v2.11
CT Analyzer	Bruker microCT (Kontich, Belgium)	v1.13.5.1
GSEA	Subramanian *et al,* 2005[1]	v3.0 [build: 0160]
Heatmapper	Babicki *et al,* 2016[2]	http://www.heatmapper.ca/
Illustrator	Adobe Systems, Inc	v21.1.0
LightCycler 480 Software	Roche	v1.5
MatLab	Mathworks	R2018b
MedCalc	MedCalc Software, Ostend, Belgium	v18.10
NRecon	Bruker microCT (Kontich, Belgium)	v1.6.9.4
PMOD	PMOD Technologies LLC (Zurich, Switzerland)	v3.806
Prism	GraphPad Software, LLC	v8.1.0 to v8.4.0
R	The R Project for Statistical Computing	v2.15.0

OTHER		
TSE Phenomaster	TSE	PhenoMaster 1.0 with software version 6.1.9
OneTouch Verio Glucometer	OneTouch	User's manual here
Gamma counter	PerkinElmer (USA)	Wizzard[2]
Preclinical PET/CT scanner	Mediso (Budapest, Hungary)	nanoScan PET/CT 122S
Clinical PET/CT scanner	Siemens Medical Systems (Erlangen, Germany	Biograph mCT
MRI scanner	Siemens Medical Systems (Erlangen, Germany	3T Verio
Clinical CT scanner	Toshiba Medical Systems (Japan)	Aquilion ONE

References cited in Supplementary Table 1:

1. Subramanian, A. *et al.* Gene set enrichment analysis: a knowledge-based approach for interpreting genome-wide expression profiles. *Proc. Natl. Acad. Sci. U. S. A.* **102**, 15545-15550 (2005).
2. Babicki, S. *et al.* Heatmapper: web-enabled heat mapping for all. *Nucleic Acids Res.* **44**, W147-153 (2016).

Supplementary Figure 1

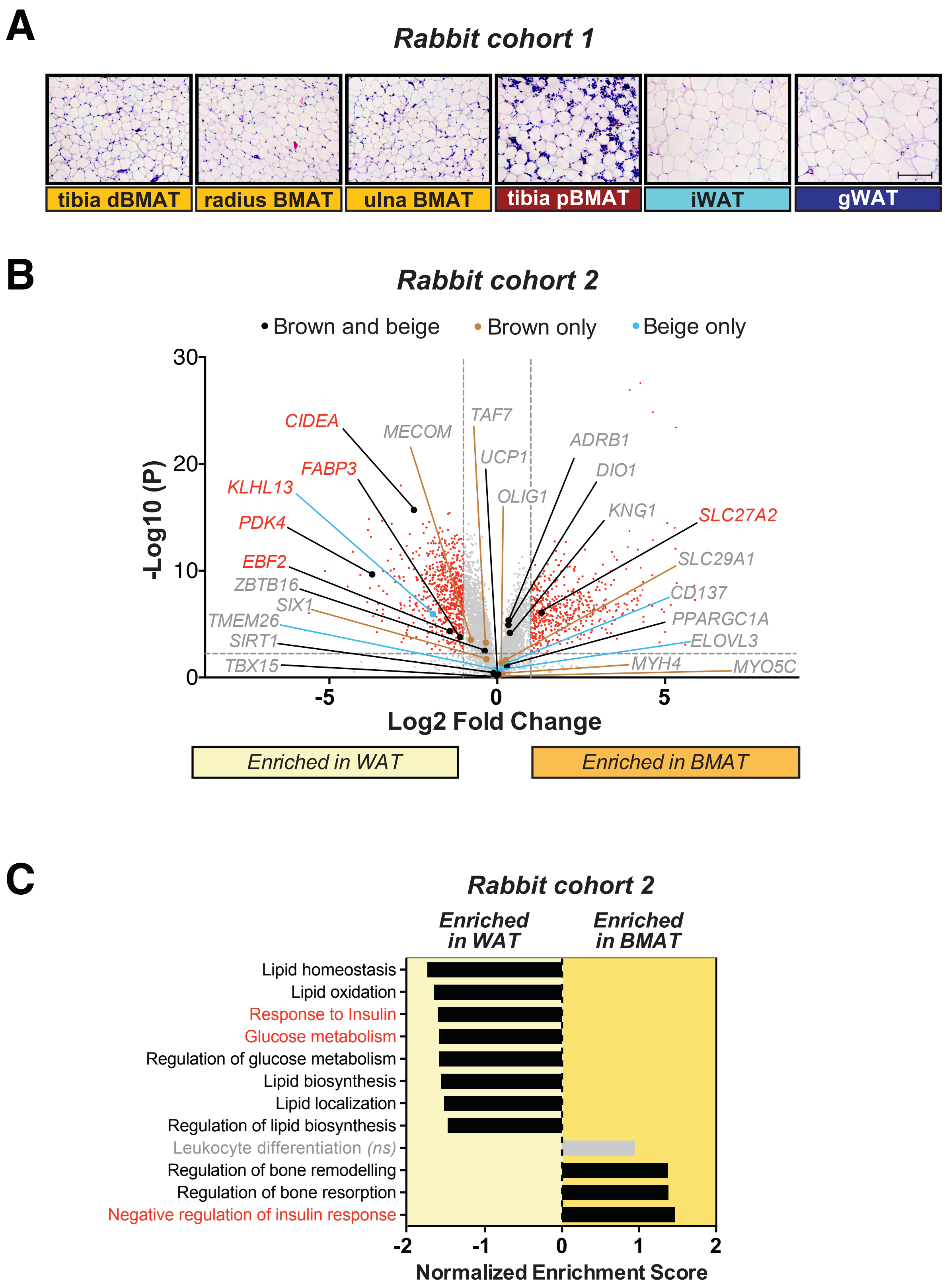

Supplementary Figure 1, Related to Figure 1 – BMAT is transcriptionally distinct from white, brown and beige adipose tissues. (A) Micrographs of BMAT and WAT isolated from rabbit cohort 1, representative of 6 rabbits. Scale bar = 150 μm. **(B-C)** Transcriptional profiling of WAT (gonadal + inguinal) vs whole BMAT (dBMAT + ruBMAT) from rabbit cohort 2. (B) Volcano plot of differentially expressed transcripts (FDR < 0.05, fold-change > 2). Transcripts characteristic of brown and/or beige adipocytes are labelled. Those with significant differential expression between WAT and BMAT are shown in red; those not differentially expressed are in grey. (C) Gene set enrichment analysis (GSEA) highlights lipid metabolism, glucose metabolism and insulin responsiveness as key pathways differentially regulated between WAT and BMAT. Source data are provided as a Source Data file.

Supplementary Figure 2

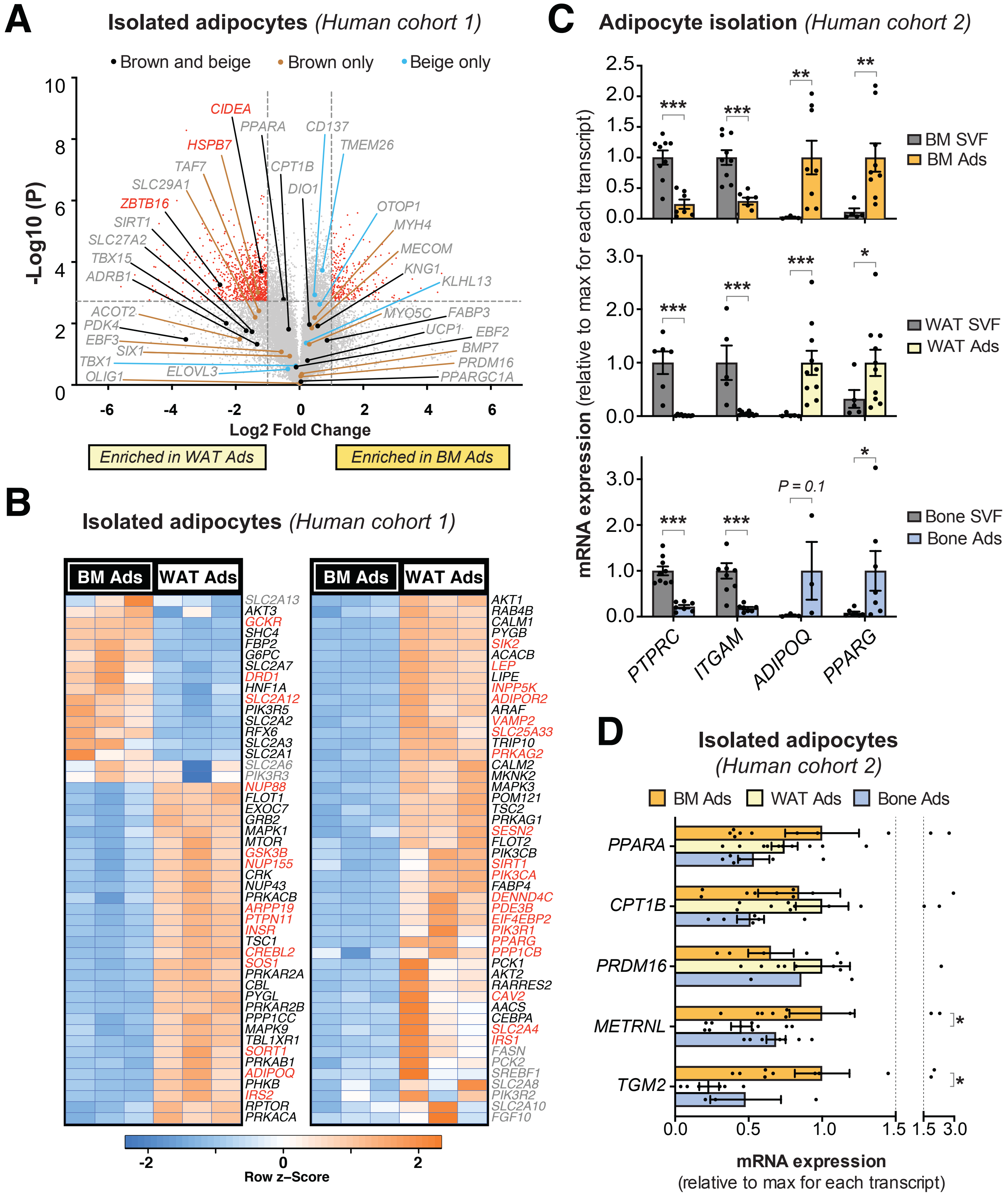

Supplementary Figure 2, Related to Figure 1 – BM adipocytes in humans are transcriptionally distinct from WAT adipocytes. Transcriptional profiling **(A,B)** and qPCR analysis **(C,D)** of adipocytes isolated from the subcutaneous WAT or femoral diaphyseal BM of humans undergoing hip-replacement surgery. (A,B) Volcano plots (A) and heat maps (B) are presented as for Figures 1 and S1. (C,D) qPCR to validate purity of adipocytes isolated from each tissue (C) and showing that BM adipocytes generally do not have increased expression of brown or beige adipocyte markers (D). In (C), *PTPRC* encodes CD45 and *ITGAM* encodes the macrophage marker CD11b. Transcript expression was normalised to expression of *IPO8* or *RNA18SN5*. Data in (C) are mean ± SEM of the following numbers of human subjects per cell type: BM Ads, n = 7 (*PTPRC, ITGAM*) 8 (*ADIPOQ)* or 9 (*PPARG*); BM SVF, n = 9 (*PTPRC, ITGAM),* 3 (*ADIPOQ*) or 5 (*PPARG)*; WAT Ads, n = 10 (*each transcript)*; WAT SVF, n = 6 (*PTPRC)* or 5 (*ITGAM, ADIPOQ* or *PPARG*); Bone Ads, n = 7 (*PTPRC, ITGAM, PPARG*) or 3 (*ADIPOQ);* Bone SVF, n = 9 (*PTPRC, ITGAM), 7* (*PPARG)* or 3 (*ADIPOQ)*. Data in (D) are mean ± SEM of the following numbers of human subjects per cell type: BM Ads, n = 10 (*PPARA, TGM2*), 9 (*CPT1B, METRNL*) or 5 *(PRDM16*); WAT Ads, n = 10 (*PPARA, CPT1B, METRNL*), 9 (*PRDM16*) or 6 (*TGM2*); Bone Ads, n = 7 (*METRNL*), 6 (*PPARA, CPT1B*), 3 *(TGM2*) or 2 (*PRDM16*). For each transcript in (C), significant differences between Ads and SVF are indicated by * (*P* <0.05), ** (*P* <0.01) or *** (*P* <0.001). Significance for normally distributed transcripts (*PTPRC* and *ITGAM* in each tissue; *ADIPOQ* in BM and Bone) was assessed by two-tailed Welch's t-test with Holm-Sidak adjustment for multiple comparisons, while non-normally distributed transcripts (*ADIPOQ* in WAT; *PPARG* in each tissue) were assessed by the two-tailed Mann-Whitney test. For each transcript in (D), significant differences between WAT Ads and BMAds or Bone Ads are indicated by * (*P* <0.05). Significance for normally distributed transcripts (*METRNL, TGM2*) was assessed by one-way ANOVA with Dunnett's test for multiple comparisons, while non-normally distributed transcripts (*PPARA, CPT1B, PRDM16*) were assessed by the Kruskal-Wallis test with Dunn's test for multiple comparisons. Source data are provided as a Source Data file.

Supplementary Figure 3

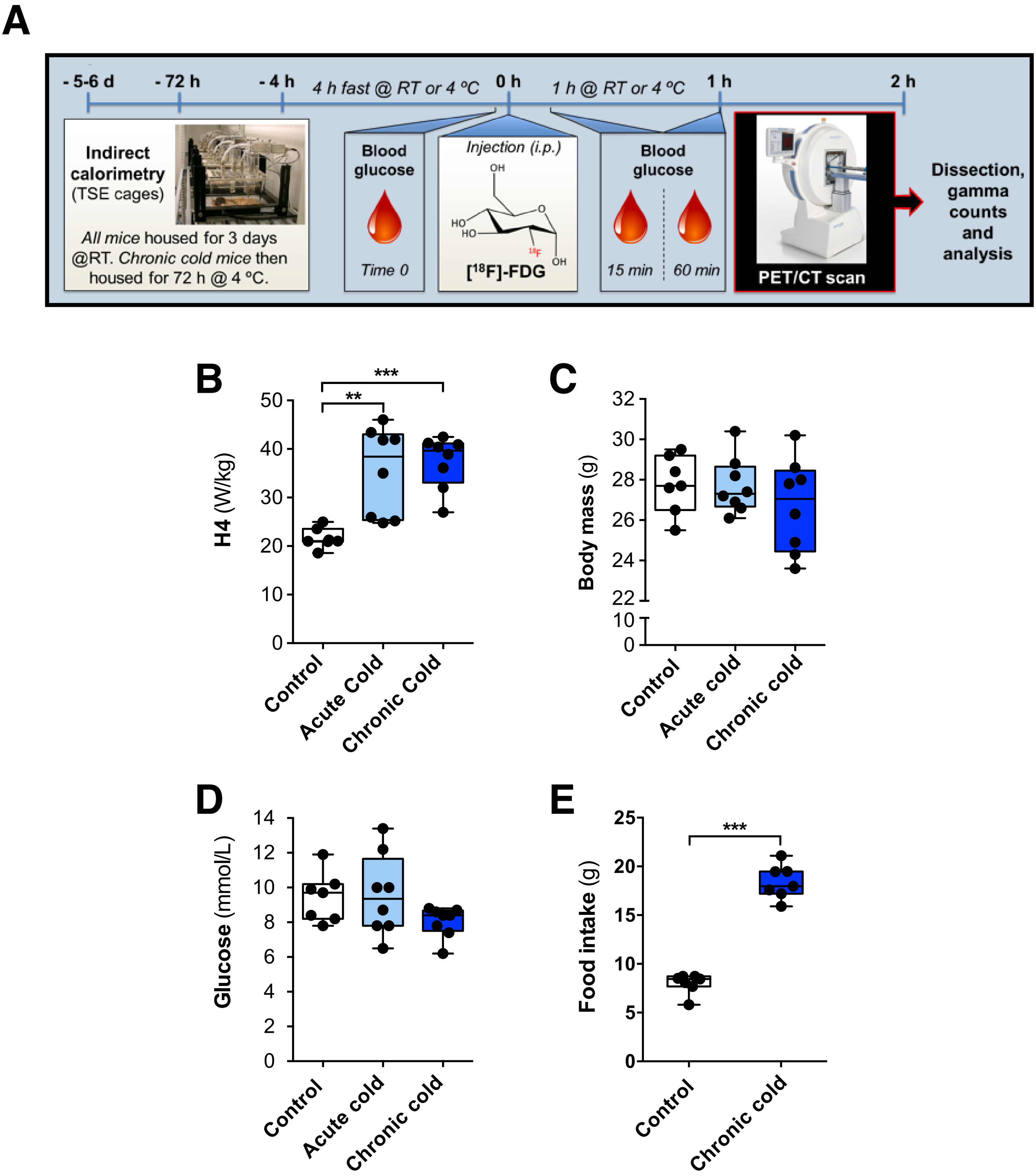

Supplementary Figure 3, Related to Figure 5 – Effects of cold exposure on energy homeostasis and gene expression in BAT, iWAT and bone. (A) Protocol for cold exposure and calorimetry studies, as described in the Methods. **(B)** Effects of cold exposure on energy expenditure (H4, W/kg), which is calculated by measuring the energy produced (H3, watts) during the light cycle (7 am to 7 pm) and dividing this by the mouse's lean body mass (kg). **(C-E)** Effects of cold exposure on body mass (C), baseline blood glucose (D) and 72 h food intake (E). Data are shown as box-and-whisker plots of 7 *Control,* 8 *Acute cold* and 8 *Chronic cold* mice per group, except in (E), where n=7 for *Chronic cold* mice; boxes indicate the 25th and 75th percentiles; whiskers display the range; and horizontal lines in each box represent the median. Significant differences between groups are indicated by ** (*P* <0.01) or *** (*P* <0.001) and were assessed as follows: (B), one-way ANOVA with Dunnet's test for multiple comparisons; (C,D), one-way ANOVA with Tukey's test for multiple comparisons; (E), two-tailed Mann-Whitney test. Source data are provided as a Source Data file.

Supplementary Figure 4

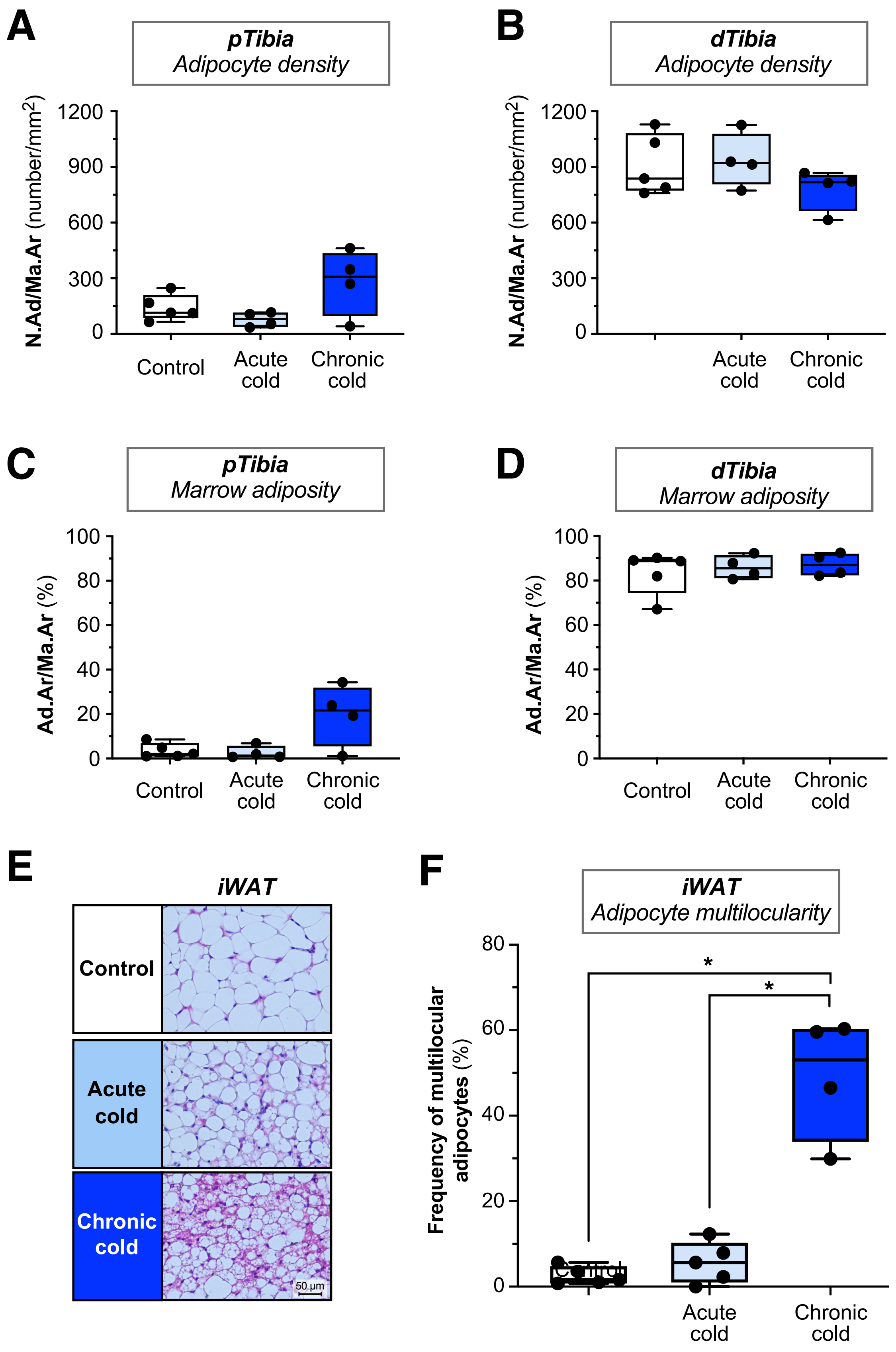

Supplementary Figure 4, Related to Figure 5 – Effects of cold exposure on BM adiposity and adipocyte morphology in iWAT and bone. Micrographs of proximal tibiae, distal tibia and iWAT from control, acute cold and chronic cold mice were analysed using Image J for morphometric analyses. **(A-D)** Adipocyte density (N.Ad/Ma.Ar, number/mm^2) and BM adiposity (Ad.Ar/Ma.Ar, %) were determined for proximal and distal tibial BM, as indicated. **(E)** Representative micrographs of H&E-stained iWAT, shown at higher magnification than in Figure 5D, in which abundant multilocular beige adipocytes are apparent in chronic cold-exposed mice; scale bar = 50 µm. **(F)** The frequency of multilocular adipocytes in iWAT as % of total adipocyte number. Data in (A-D) and (F) are shown as box-and-whisker plots of 5 (*Control*) or 4 (*Acute cold, Chronic cold*) mice per group; boxes indicate the 25th and 75th percentiles; whiskers display the range; and horizontal lines in each box represent the median. In (A-D), no significant differences were detected between groups using one-way ANOVA with Tukey's test for multiple comparisons (A,B,D) or the Kruskal-Wallis test with Dunn's test for multiple comparisons (C). In (F), significant differences between groups are indicated by * (P <0.05) and were assessed using Welch's one-way ANOVA with Dunnett's T3 test for multiple comparisons. Source data are provided as a Source Data file.

Supplementary Figure 5

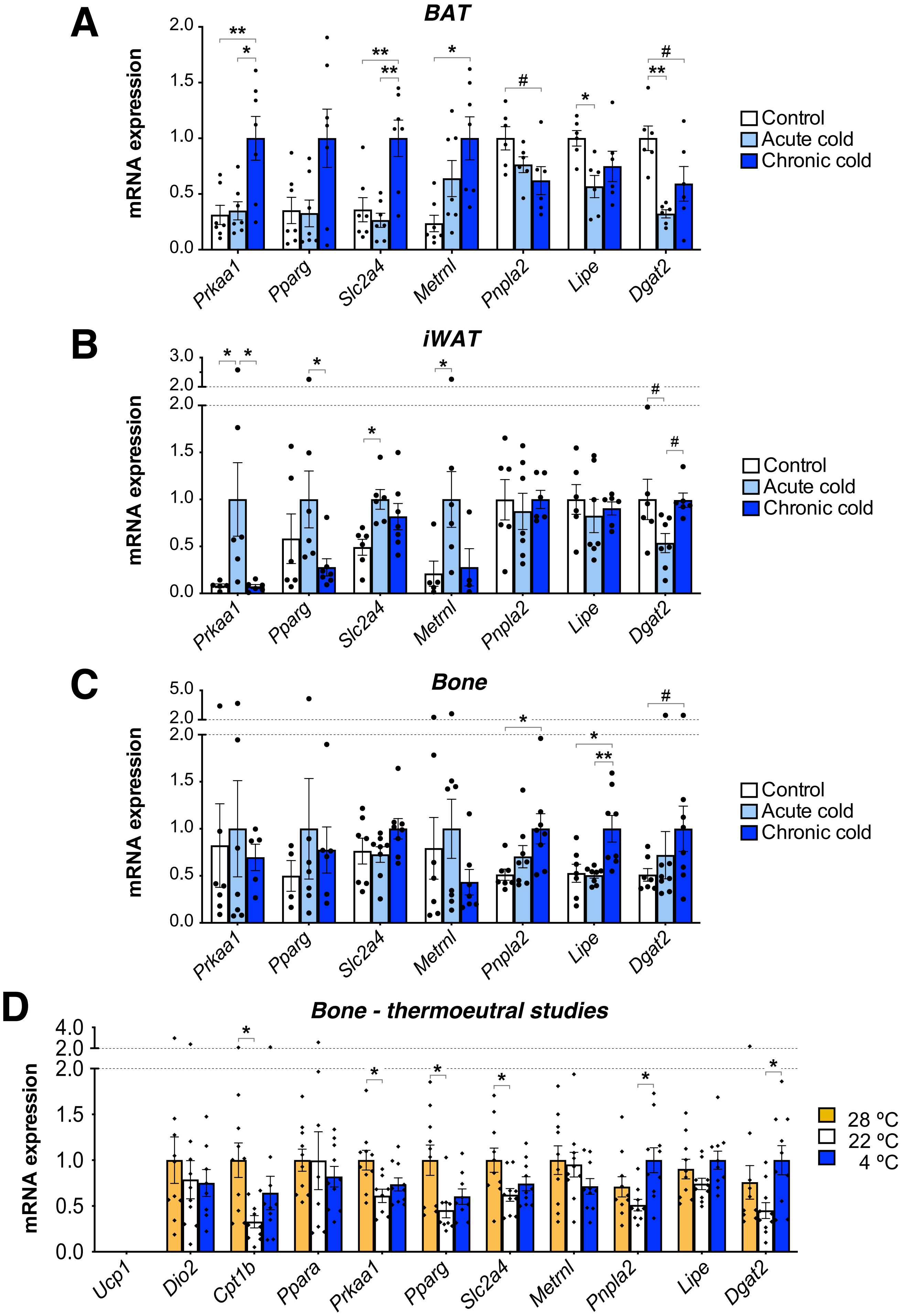

Supplementary Figure 5, Related to Figure 5 – Effects of cold exposure on transcript expression in BAT, iWAT and bone. Total RNA was isolated from BAT, iWAT or whole femurs of control, acute cold and chronic cold mice **(A-C)** or from whole femurs from a separate cohort of mice that were housed for 48 h at thermoneutrality, 22ºC or 4ºC **(D)**. Expression of BAT or beige cold exposure markers (*Prkaa1, Pparg, Slc2a4, Metrnl*), lipases (*Pnpla2, Lipe*) or lipogenic factors (*Dgat2*) was then determined by qPCR; *ND* = not detectable. Transcript expression was normalised to the geometric mean of *Rn18s* and *Ppia* expression (A,C,D) or *Ppia* only (B); the latter was used for iWAT because in this tissue *Rn18s,* but not *Ppia,* showed significant regulation between the three groups. For each tissue in (A-D) data are shown as mean ± SEM of the following numbers of mice per group: BAT (*Control, Acute cold* or *Chronic cold*)*,* n=7 (*Prkaa1, Pparg, Slc2a4, Metrnl*) or 6 (*Pnpla2, Lipe, Dgat2*); iWAT *(Control*)*,* n=5 (*Prkaa1, Metrnl*) or 6 (*Pparg, Slc2a4, Pnpla2, Lipe, Dgat2*); iWAT (*Acute cold*), n=6 (*Prkaa1, Pparg, Slc2a4, Metrnl*) or 7 (*Pnpla2, Lipe, Dgat2*); iWAT (*Chronic cold*), n=6 (*Prkaa1, Pnpla2, Lipe, Dgat2*), 7 (*Pparg, Slc2a4*) or 4 (*Metrnl*); Bone (*Control*), n= 7 (*Prkaa1, Slc2a4, Metrnl*, *Pnpla2, Lipe, Dgat2*) or 4 (*Pparg*); Bone (*Acute cold*), n=7 (*Prkaa1, Pparg*) or 8 (*Slc2a4, Metrnl*, *Pnpla2, Lipe, Dgat2*); Bone (*Chronic cold*), n=5 (*Prkaa1*), 6 (*Pparg*), 7 (*Metrnl*) or 8 (*Slc2a4, Pnpla2, Lipe, Dgat2*); Bone (28ºC), n=10 (*each transcript);* Bone (22ºC), n=10 (*Ucp1, Dio2, Cpt1b, Slc2a4, Metrnl, Lipe, Dgat2*), 9 (*Pparg, Pnpla2*) or 8 (*Ppara, Prkaa1*); Bone (4ºC), n=10 (*Ucp1, Cpt1b, Ppara, Prkaa1, Slc2a4, Metrnl, Pnpla2, Lipe, Dgat2*), 8 (*Dio2*) or 9 *(Pparg*). For each transcript, significant differences between groups are indicated by # (P <0.1), * (P <0.05), ** (P <0.01) or *** (P <0.001). The following transcripts are non-normally distributed and were assessed using the Kruskal-Wallis test with Dunn's test for multiple comparisons: (A), *Prkaa1, Pparg;* (B), *Pparg, Metrnl*; (C), *Prkaa1, Metrnl*; (D), *Dio2, Cpt1b, Pparg, Dgat2.* Data for all other transcripts are normally distributed and were assessed using one-way ANOVA with Dunnet's or Tukey's tests for multiple comparisons. Source data are provided as a Source Data file.

Supplementary Figure 6

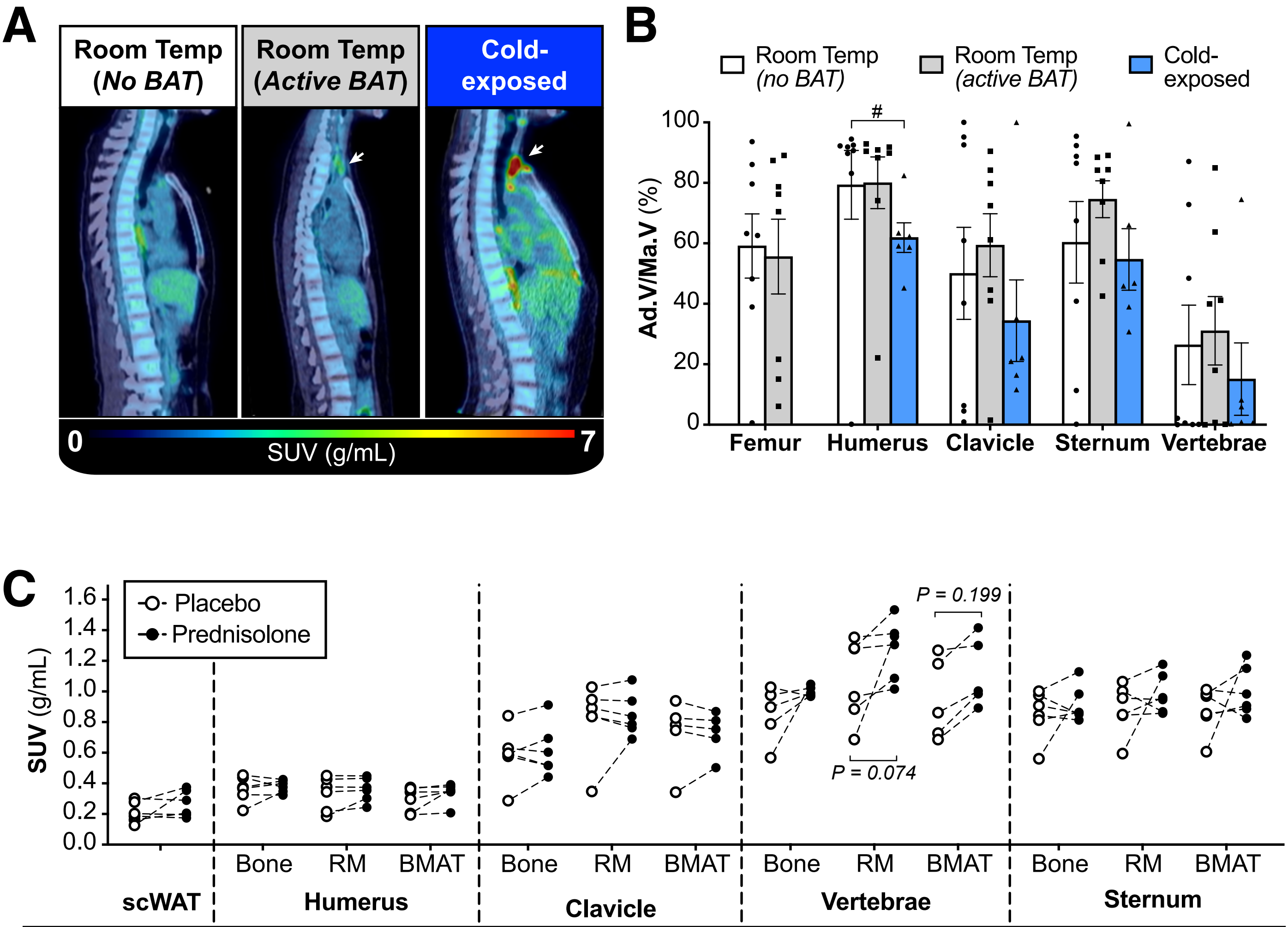

	Humerus	Clavicle	Vertebrae	Sternum
**Treatment**	*P* = 0.0531	*P* = 0.5116	***P* = 0.0037**	*P* = 0.0989
**Tissue**	*P* = 0.4467	*P* = 0.1111	*P* = 0.1213	*P* = 0.5374
**Interaction**	*P* = 0.7581	*P* = 0.8844	*P* = 0.8996	*P* = 0.9213

Supplementary Figure 6, Related to Figure 7 – Human BMAT is functionally distinct from BAT. (A) Representative sagittal PET/CT scans highlighting the BM cavities of the vertebrae and sternum; arrows indicate ^{18}F-FDG uptake in supraclavicular BAT. **(B)** Quantification of BMAT in CT scans of the *No BAT, Active BAT* and *Cold* subjects, as done for the <60 and >60 groups in Figure 6E. Data are shown as mean ± SEM of 8 (*No BAT*) or 7 (*Active BAT, Cold)* subjects per group. Within each tissue, significant differences between groups are indicated by # (*P* <0.05) and were assessed using a two-tailed Mann-Whitney test (Femur) or the Kruskal-Wallis test with Dunn's test for multiple comparisons (Humerus, Clavicle, Sternum, Vertebrae). **(C)** Subjects were treated with prednisolone or placebo control prior to analysis of ^{18}F-FDG uptake by PET/CT. For each tissue, data are shown as paired individual values from 6 subjects, except for clavicular bone, vertebral bone and vertebral BMAT, where only 5 subjects could be included. For each skeletal site, the influence of treatment or tissue (bone, RM, BMAT), and interactions between these, were determined by 2-way ANOVA; *P* values are shown beneath the graph. Source data are provided as a Source Data file.
