# Supplementary material for: Bone marrow adipose tissue is a unique adipose subtype with distinct roles in glucose homeostasis
Source: Nat Commun. 2020 Jun 18;11:3097. doi: 10.1038/s41467-020-16878-2 (PMC7303125; doi:10.1038/s41467-020-16878-2)
Supplement: Supplementary file 3 — Reporting Summary [file 41467_2020_16878_MOESM3_ESM.pdf]

nature research

Corresponding author(s): William Cawthorn
Last updated by author(s): Nov 21, 2019

Reporting Summary

Nature Research wishes to improve the reproducibility of the work that we publish. This form provides structure for consistency and transparency in reporting. For further information on Nature Research policies, see Authors & Referees and the Editorial Policy Checklist .

Statistics

For all statistical analyses, confirm that the following items are present in the figure legend, table legend, main text, or Methods section.

n/a	Confirmed	
☐	☒	The exact sample size (*n*) for each experimental group/condition, given as a discrete number and unit of measurement
☐	☒	A statement on whether measurements were taken from distinct samples or whether the same sample was measured repeatedly
☐	☒	The statistical test(s) used AND whether they are one- or two-sided<br>*Only common tests should be described solely by name; describe more complex techniques in the Methods section.*
☒	☐	A description of all covariates tested
☐	☒	A description of any assumptions or corrections, such as tests of normality and adjustment for multiple comparisons
☐	☒	A full description of the statistical parameters including central tendency (e.g. means) or other basic estimates (e.g. regression coefficient) AND variation (e.g. standard deviation) or associated estimates of uncertainty (e.g. confidence intervals)
☐	☒	For null hypothesis testing, the test statistic (e.g. *F*, *t*, *r*) with confidence intervals, effect sizes, degrees of freedom and *P* value noted<br>*Give P values as exact values whenever suitable.*
☒	☐	For Bayesian analysis, information on the choice of priors and Markov chain Monte Carlo settings
☒	☐	For hierarchical and complex designs, identification of the appropriate level for tests and full reporting of outcomes
☒	☐	Estimates of effect sizes (e.g. Cohen's *d*, Pearson's *r*), indicating how they were calculated

Our web collection on statistics for biologists contains articles on many of the points above.

Software and code

Policy information about availability of computer code

Data collection

(1) PMOD v3.806 (PMOD Technologies LLC, Zurich, Switzerland)), a commercial PET-CT image analysis software, was used to perform image analysis and extract values from the PET-CT imaging data.

(2) For quantitative PCR (qPCR), data were collected using LightCycler 480 Software, v1.5 (Roche)

(3) Data from µCT scans were reconstructed using NRecon v1.6.9.4 software (Bruker, Kontich, Belgium).

Data analysis

(1) To prepare the test and classification variables on a per pixel basis for ROC analysis, a customised in-house software was developed in Matlab version R2018b (MathWorks Inc., Natick, MA, USA). ROC analysis was performed in MedCalc version 18.10 (MedCalc Software, Ostend, Belgium). Code for the in-house ROC analysis has been deposited in GitHub and is available at https://github.com/Georgerun/ROCPerPixel.

(2) Transcript expression from qPCR was determined using LightCycler 480 Software, v1.5 (Roche).

(3) Volumetric analysis of µCT data was performed using CT Analyzer v1.13.5.1 (Bruker microCT, Kontich, Belgium).

(4) 3D renders of µCT and PET/CT data were generated using Analyze v12.0 (AnalyzeDirect, Overland Park, KS, USA).

(5) Microarray data were analysed as follows:
(5.i) Affy, affyPLM, and limma packages of Bioconductor (v2.11), implemented in the R-statistical environment (v2.15.0), were used to analyse the data, including PCA analysis. The Affy package was used to fit log2 expression values to the data. The limma package was used to fit and contrast-compute weighted, paired, linear models to the data.

(5.ii) Microarrays were analysed for differential gene expression using Gene Set Enrichment Analysis software v3.0 [build: 0160] and the

Molecular Signature Database (MSigDB, v6.2) (Subramanian, Tamayo, et al. (2005), PNAS 102, 15545-15550, http://www.broad.mit.edu/gsea/).

(6) Heatmaps of differentially expressed genes were generated using Heatmapper software; no version number is available, but further details are available at http://www.heatmapper.ca/

(7) Data visualisation (e.g. graph drawing) and statistical analysis was done using Prism v.8.1.0 to v8.4.0 (GraphPad Software, LLC).

(8) Final versions of figures were generated using Adobe Illustrator v21.1.0 (Adobe Systems, Inc).

For manuscripts utilizing custom algorithms or software that are central to the research but not yet described in published literature, software must be made available to editors/reviewers. We strongly encourage code deposition in a community repository (e.g. GitHub). See the Nature Research guidelines for submitting code & software for further information.

Data

Policy information about availability of data

All manuscripts must include a data availability statement. This statement should provide the following information, where applicable:
- Accession codes, unique identifiers, or web links for publicly available datasets
- A list of figures that have associated raw data
- A description of any restrictions on data availability

All relevant data are available from the authors upon reasonable request. The source data underlying Figs 1B-E, 1G, 2A-B, 3A, 3C, 3E-F, 4A-B, 5B-C, 5E-G, 6B, 6E, 7B-F, S2C-D, S3B-E, S4A-D, S4F, S5A-D and S6B-C are provided as a Source Data file. Microarray data for analysis of rabbit cohorts 1 and 2 has been made publicly available on the NCBI GEO platform73 under the series ID GSE138690 (https://www.ncbi.nlm.nih.gov/geo/query/acc.cgi?acc=GSE138690).

The Molecular Signature Database (MSigDB, v6.2), used for Gene Set Enrichment Analysis, can be accessed at https://www.gsea-msigdb.org/gsea/msigdb/collections.jsp

Field-specific reporting

Please select the one below that is the best fit for your research. If you are not sure, read the appropriate sections before making your selection.

☒ Life sciences ☐ Behavioural & social sciences ☐ Ecological, evolutionary & environmental sciences

For a reference copy of the document with all sections, see nature.com/documents/nr-reporting-summary-flat.pdf

Life sciences study design

All studies must disclose on these points even when the disclosure is negative.

Sample size	Sample sizes to assess effects of insulin treatment (n = 5-7 per group) or cold exposure (n = 7-10 per group) were based on previous studies that have assessed insulin-stimulated glucose uptake (Zoch et al, 2016, PMID 27088042) or effects of cold exposure on glucose uptake or beiging of adipose tissues (Wang et al, 2012, PMID 23207798; Fabbiano et al, 2016, PMID 27568549; Jeanguillaume et al. 2013, DOI:10.4236/ami.2013.33004).
Data exclusions	No data was excluded from the analysis.
Replication	Mouse PET/CT was split into two cohorts for the insulin-treatment studies and three cohorts for the cold-exposure studies. Each replication was successful and no cohort-specific effects were observed.
Randomization	For analysis of the effects of insulin treatment in mice (Fig. 3) or rats (Fig. 4), or cold-exposure in mice (Figs. 5, S3-S5), animals were randomly assigned to each group (Mice: chronic cold, acute cold, room temperature; insulin and saline. Rats: insulin and saline). For comparison of gene or protein expression between BMAT and WAT of rabbits (Fig. 1, S1) or rats (Fig. 2), there were no experimental interventions and therefore randomisation was not applicable. Cold-exposed humans were part of a randomized study (Ramage, 2016). Subjects with or without active BAT at room temperature were identified retrospectively from a database of PETCT scans. Co-variates were not accounted for in our analysis. Subjects were matched for age, BMI, and disease status to minimize effects of these potential covariates.
Blinding	Human PETCT analysis was blinded by the use of randomly generated patient IDs. Animal work was blinded at point of analysis, but blinding was not possible during cold housing and insulin treatment. This is because, for cold-exposure, mice were housed in TSE Phenomaster cages at 4ºC, during which they were housed separately to the room temperature controls. For insulin treatment, blinding was not possible because the robust effects of insulin on blood glucose (Fig. 3A) and myocardial FDG uptake (Fig. 3B) clearly distinguish the insulin-treated vs the saline-treated mice. However, all downstream analysis was done under blinded conditions.

Reporting for specific materials, systems and methods

We require information from authors about some types of materials, experimental systems and methods used in many studies. Here, indicate whether each material, system or method listed is relevant to your study. If you are not sure if a list item applies to your research, read the appropriate section before selecting a response.

Materials & experimental systems

n/a	Involved in the study
☐	☒ Antibodies
☒	☐ Eukaryotic cell lines
☒	☐ Palaeontology
☐	☒ Animals and other organisms
☐	☒ Human research participants
☐	☒ Clinical data

Methods

n/a	Involved in the study
☒	☐ ChIP-seq
☒	☐ Flow cytometry
☐	☒ MRI-based neuroimaging

Antibodies

Antibodies used

Primary antibodies: Insulin Receptor-β (Santa Cruz, sc-711), IRS-1 (Upstate, 06-248), GLUT4 (Cell Signaling, 2213), Adiponectin (Sigma, A6354), ERK1/2 (Cell Signaling, 9102), P-Akt S473 (Cell Signaling, 9271), P-Akt T308 (Santa Cruz, sc-16646), Akt (Cell Signaling, 4691), alpha-Tubulin (Pierce, 80017).

Secondary antibodies (HRP-linked): anti-rat IgG (GE Healthcare, NA935), anti-rabbit IgG (GE Healthcare, NA934).

Validation

Each primary antibody has been used as recommended by the manufacturer and as cited extensively in previous publications. We further ensured that signals in Western Blots were present at the expected size for each target and the kDa for each band is shown in our manuscript. The relevant information for each primary antibody, from the manufacturer and online databases, is as follows:

Insulin Receptor-β (Santa Cruz, sc-711): recommended by the manufacturer for detection of Insulin Receptor-β of mouse, rat and human origin by Western Blotting, immunoprecipitation, immunofluorescence, and solid-phase ELISA (https://datasheets.scbt.com/sc-711.pdf); 382 citations on the CiteAb database (https://www.citeab.com/antibodies/801523-sc-711-insulin-r-antibody-c-19).

IRS-1 MilliporeSigma, 06-248): routinely validated by the manufacturer against lysates of NIH3T3 cells and recommended for detection of IRS-1 of mouse, rat and human origin by Western Blotting and immunoprecipitation (https://www.sigmaaldrich.com/catalog/product/mm/06248); 178 citations on the CiteAb database (https://www.citeab.com/antibodies/220328-06-248-anti-irs1-antibody).

GLUT4 (Cell Signaling, 2213): recommended by the manufacturer for detection of GLUT4 of mouse, rat and human origin by Western Blotting, with specificity confirmed for endogenous GLUT4 and no cross-reactivity with other related proteins (https://www.cellsignal.co.uk/products/primary-antibodies/glut4-1f8-mouse-mab/2213); 72 citations on the CiteAb database (https://www.citeab.com/antibodies/123042-2213-glut4-1f8-mouse-mab).

Adiponectin (Sigma, A6354): recommended by the manufacturer for detection of adiponectin of mouse and human origin by Western Blotting and immunohistochemistry, and used in at least 23 cited papers (https://www.cellsignal.co.uk/products/primary-antibodies/glut4-1f8-mouse-mab/2213). As shown in Figure 2 and in the Source Data file, our Western Blots confirm that this antibody detects a band at 30 kDa, as expected for adiponectin, in lysates of WAT and BMAT, but not in lysates of red marrow. This validates the specificity of this antibody for detection of adiponectin in rat lysates.

ERK1/2 (Cell Signaling, 9102): recommended by the manufacturer for detection of ERK1/ERK2 of mouse, rat, human, hamster, monkey, mink, zebrafish, bovine, pig, or yeast (S. cerevisiae) origin by Western Blotting, immunohistochemistry and immunoprecipitation, with specificity confirmed for endogenous ERK1/ERK2 and no cross-reactivity with other related proteins (JNK/SAPK or p38 MAP kinase) (https://www.cellsignal.co.uk/products/primary-antibodies/p44-42-mapk-erk1-2-antibody/9102). Over 4,000 citations on the CiteAb database (https://www.citeab.com/antibodies/125750-9102-p44-42-mapk-erk1-2-antibody).

P-Akt S473 (Cell Signaling, 9271): recommended by the manufacturer for detection of Phospho-Akt (Ser473) of mouse, rat, human, hamster, fruit fly, bovine, dog, and pig origin by Western Blotting, immunofluorescence, immunoprecipitation and flow cytometry; specificity has been confirmed for endogenous levels of Akt1 only when phosphorylated at Ser473, and this antibody also recognizes Akt2 and Akt3 when phosphorylated at the corresponding residues. It does not recognize Akt phosphorylated at other sites, nor does it recognize phosphorylated forms of related kinases such as PKC or p70 S6 kinase (https://www.cellsignal.co.uk/products/primary-antibodies/p44-42-mapk-erk1-2-antibody/9102). Over 4,000 citations on the CiteAb database (https://www.citeab.com/antibodies/125972-9271-phospho-akt-ser473-antibody).

P-Akt T308 (Santa Cruz, sc-16646-R): recommended by the manufacturer for detection of Insulin Receptor-β of mouse, rat and human origin by Western Blotting, immunoprecipitation, immunofluorescence, immunohistochemistry and solid-phase ELISA (https://datasheets.scbt.com/sc-16646.pdf); 151 citations on the CiteAb database (https://www.citeab.com/antibodies/3430458-sc-16646-r-p-akt1-2-3-antibody-thr-308-r).

Akt (Cell Signaling, 4691): recommended by the manufacturer for detection of total Akt of mouse, rat, human, fruit fly and monkey origin Western Blotting, immunoprecipitation, immunofluorescence, immunohistochemistry and flow cytometry; specificity has been confirmed for endogenous levels of total Akt, with no cross-reactivity for other related proteins (https://www.cellsignal.co.uk/products/primary-antibodies/akt-pan-c67e7-rabbit-mab/4691). Over 1,900 citations on the CiteAb

database (https://www.citeab.com/antibodies/124655-4691-akt-pan-c67e7-rabbit-mab).

alpha-Tubulin (Invitrogen, MA1-80017): recommended by the manufacturer for detection of alpha-tubulin of mouse, rat, human, rabbit, pig, dog, fruit fly, Xenopus and yeast origin by Western Blotting, immunocytochemistry, immunofluorescence and ELISA, and as a suitable loading control in Western Blots (https://www.thermofisher.com/antibody/product/alpha-Tubulin-Antibody-clone-YL1-2-Monoclonal/MA1-80017); 32 citations on the CiteAb database (https://www.citeab.com/antibodies/88028-ma1-80017-alpha-tubulin-monoclonal-antibody-yl1-2).

Animals and other organisms

Policy information about studies involving animals; ARRIVE guidelines recommended for reporting animal research

Laboratory animals

Male Rabbits - New Zealand White rabbits (13 and 22 weeks of age). Male mice - C57BL/6J (16 weeks of age). Male rats – Sprague-Dawley (13-15 weeks of age). Female rats – high-capacity and low-capacity runner rats (one year of age).

Wild animals

The study did not involve wild animals.

Field-collected samples

The study did not involve field-collected samples.

Ethics oversight

Studies in New Zealand White rabbits and rats were approved by the University of Michigan Committee on the Use and Care of Animals, with daily care overseen by the Unit for Laboratory Animal Medicine. Studies in C57BL/6JCrl mice were approved by the University of Edinburgh Animal Welfare and Ethical Review Board and were done under project licenses granted by the UK Home Office.

Note that full information on the approval of the study protocol must also be provided in the manuscript.

Human research participants

Policy information about studies involving human research participants

Population characteristics

Cohort 1: Subjects undergoing hip-replacement surgery or abdominal surgery (for BMAd and WAT Ad isolation, respectively; Fig. 1E, Supplementary Fig. 2A-B) are as described previously (Mattiucci et al, 2018, DOI: https://doi.org/10.1002/jcp.26037).

Cohort 2: Subjects undergoing hip-replacement surgery (for isolation of BMAds and WAT Ads; Fig. 1F-G, Supplementary Fig. 2C-D) included 4 males and 6 females (aged 67.1   5.9 years; BMI of 31.6   6.9).

Cohort 3: Subjects undergoing paired MRI and CT scans (Fig. 6A-C) were from the following trials:
(3.1) The SALTIRE 2 trial (Bisphosphonates and RANKL Inhibition in Aortic Stenosis, NCT02132026) recruited patients aged over 50 years with a peak aortic jet velocity of >2.5 m/s and grade 2-4 calcification of the aortic valve on echocardiography.
(3.2) The PRE18FFIR trial (Prediction of recurrent events with 18F-fluoride to identify ruptured and high-risk coronary artery plaques in patients with myocardial infarction, NCT02278211) recruited patients with recent myocardial infarction and multi-vessel coronary artery disease on invasive angiography. Exclusion criteria for both trials include inability to receive iodinated contrast, renal impairment (estimated glomerular filtration rate ≤30 mL/min/1.73 m2) or women of child-bearing potential.
In the present manuscript, subjects from the SALTIRE 2 and PRE18FFIR trials included 24 males and 9 females (aged 65.7 +/- 8.1 years; BMI of 29.1 +/- 4.8).

Cohort 4: Cold-exposed subjects (Fig. 6E, 7A-B; Supplementary Fig. 6A-B) included seven healthy male volunteers (aged 20.9 +/- 1.9 years; BMI of 22.8 +/- 1.4) who were exposed to a mild cold (16-7ºC) for 2 h prior to their 18F-FDG PET/CT scan, as described previously (Weir et al, 2018, DOI: https://doi.org/10.1016/j.cmet.2018.04.020).

Cohort 5: Subjects with or without detectable BAT at room temperature (Fig. 6D-E, 7A-F and S6A-B of revised manuscript) and described further in Table 1 of the Methods section. 'No BAT' subjects included 2 males and 8 females (aged 51.5 +/- 19.6 years; BMI of 20.7 +/- 2.4). 'Active BAT' subjects included 2 males and 8 females (aged 51.1 +/- 16.0 years; BMI of 21.0 +/- 2.2).

Cohort 6: Subjects with placebo or prednisolone treatment (Fig. S6C of revised manuscript) included six healthy young men (aged 22.1 +/- 2.8 years; BMI of 22.0 +/- 2.1) who were recruited to a double-blind, randomised crossover study, as described previously (Ramage et al., 2016, DOI: https://doi.org/10.1016/j.cmet.2016.06.011).

Recruitment

Cohort 1: Subjects were recruited from the Orthopedic and Traumatology Department, Ospedali Riuniti (Ancona, Italy), as described previously (Mattiucci et al, 2018, DOI: https://doi.org/10.1002/jcp.26037).

Cohort 2: Subjects were recruited from the Department of Orthopaedic Surgery, Royal Infirmary of Edinburgh (Edinburgh, UK).

Cohort 3: Study subjects from two ongoing 18F-fluoride PET/CT trials were approached regarding participation and having a PET/MR scan immediately following their PET/CT. These trials are the SALTIRE 2 trial (Bisphosphonates and RANKL Inhibition in Aortic Stenosis, NCT02132026) and the PRE18FFIR trial (Prediction of recurrent events with 18F-fluoride to identify ruptured and high-risk coronary artery plaques in patients with myocardial infarction, NCT02278211). PRE18FFIR and SALTIRE 2 participants were consented for anonymised data to be used in ethically approved studies.

Cohort 4 and 6. Human participants were recruited by newspaper advert.

Cohort 5: Subjects were recruited from the Clinical Research Imaging Centre, Royal Infirmary of Edinburgh (Edinburgh, UK).

For all human studies there are no anticipated self-selection biases that are likely to impact the study results. This is because factors that might influence study participation, including demographic factors (e.g. socioeconomic status, ethnicity, educational attainment) and pschyosocial factors (e.g. personality, behavioural traits), have not been reported to and/or are not expected to alter BMAT physiology.

Ethics oversight

All human studies (cohorts 1-6) were done in compliance with all relevant ethical regulations; following approval by the relevant local and/or national ethics committees; and in accordance with the Declaration of Helsinki, with all patients providing written informed consent prior to any study procedures.

Cohort 1: These studies were approved the local ethical committee (300/DG) of the Università Politecnica delle Marche-Azienda Ospedali Riuniti (Ancona, Italy), as described previously (Mattiucci et al, 2018, DOI: https://doi.org/10.1002/jcp.26037).

Cohorts 2-6: Studies in human cohorts 2, 3, 4 and 6 were reviewed and approved by the South East Scotland Research Ethics Committee (REC). The study in human cohort 5 was approved by the Caldicott Guardian for NHS Lothian. Ethical details specific to each study are described below:

Cohort 2: These studies were assigned REC number 10/S1102/39 and were further approved by the Academic and Clinical Central Office for Research and Development (ACCORD).

Cohort 3: Subjects underwent paired MRI and CT scans as part of the SALTIRE 2 trial (NCT02132026) or PRE18FFIR trial (NCT02278211), in which participants were consented for anonymised data to be used in ethically approved studies. Only baseline measurements not related to the trial outcomes, and not any of the pre-specified endpoints from the PRE18FFIR and SALTIRE 2 trials, are reported herein. These paired MRI-CT studies were assigned REC number 16/SS/0166 and were further approved by the United Kingdom (UK) Administration of Radiation Substances Advisory Committee.

Cohorts 4-6: These PET/CT studies were further reviewed and approved by the University of Edinburgh and NHS Lothian Academic and Clinical Central Office for Research and Development (ACCORD). Studies in cold-exposed subjects (Cohort 4) were assigned REC number 13/SS/0242. Studies in Cohort 5 were assessed and approved by The Caldicott Guardian for NHS Lothian. Studies involving placebo or prednisolone treatment (Cohort 6) were assigned REC number 11/SS/0074 and were not considered a CTIMP (clinical trial of an investigational medicinal product) because prednisolone was given to determine its physiological effect on BAT activation, as opposed to as a treatment or diagnostic test for a medical condition. For Cohort 4, further details are available at https://www.hra.nhs.uk/planning-and-improving-research/application-summaries/research-summaries/the-role-of-brown-adipose-tissue-in-humans/

Note that full information on the approval of the study protocol must also be provided in the manuscript.

Clinical data

Policy information about clinical studies

All manuscripts should comply with the ICMJEguidelines for publication of clinical research and a completedCONSORT checklist must be included with all submissions.

Clinical trial registration

A. NCT02132026 and NCT02278211

B-D. The human studies were not classified as clinical trials so were not registered with ClinicalTrials.gov.

Study protocol

A. https://clinicaltrials.gov/ct2/show/NCT02132026 and https://clinicaltrials.gov/ct2/show/NCT02278211

B-D. Full details of the study protocols relating to the human PET/CT scans have been published previously (Ramage et al, Cell Metabolism 2016; 24:130-141, DOI: https://doi.org/10.1016/j.cmet.2016.06.011; Weir et al, Cell Metabolism 2018; 27: 1348-1355, https://doi.org/10.1016/j.cmet.2018.04.020).

Data collection

A. February 2017 to December 2017.

B-D. The studies relating to the human PET/CT data were undertaken in the Edinburgh Clinical Research Facility and the Edinburgh Imaging Facility in the Queen's Medical Research Institute between March and September 2012 (healthy volunteers) and between November 2014 and February 2016 (analysis of PET/CT scans in NHS patients).

Outcomes

A. Using Analyze 12.0 software, the MR and CT scans were co-registered and volumes of interest were manually drawn around the sternum, vertebrae and subcutaneous adipose tissue. HU were extracted on a per voxel basis, and data underwent post-processing using Matlab to measure the total number of voxels across all patient HU (Fig. 4B), prior to ROC analysis (MedCalc). ROC analysis was then conducted on per voxel HU to determine threshold values with the greatest sensitivity and specificity to detect bone, yellow marrow and red marrow. Thresholds of above 300 HU were defined as bone regions, -200 to 115 HU as yellow marrow and 115 to 300 as red marrow.

A-D. PET/CT data was analyzed using PMOD version 3.806 (PMOD, Zurich, Switzerland).

Magnetic resonance imaging

Experimental design

Design type	Resting state
Design specifications	Single 60 minute scan for each participant
Behavioral performance measures	N/A

Acquisition

Imaging type(s)	Structural
Field strength	3 Tesla
Sequence & imaging parameters	All data were acquired using a 3T Verio system (Siemens AG, Healthcare Sector, Erlangen, Germany). Native T1 relaxation rates (i.e. in the absence of contrast agents) were calculated using the modified Look-Locker inversion (MOLLI) recovery technique. Perfusion images were acquired using a turbo-fast low angle shot (FLASH) saturation recovery prepared single-shot gradient echo pulse sequence (repetition time/ echo time 2.20 ms/1.07 ms, flip angle 12o, slice thickness 8 mm, preparation pulse delay (PD) to central line of k-space 100 ms, matrix size 192 × 108 and FoV 330 mm × 440 mm). With the application of GRAPPA (accelerator factor of 3) and partial Fourier acquisition of 0.75, each dynamic frame consisted of 48-phase encoded lines.
Area of acquisition	Chest

Diffusion MRI ☐ Used ☒ Not used

Preprocessing

Preprocessing software	Siemens E7 tools
Normalization	Further information: please see Papanastasiou et al, 2015, DOI:10.1186/s12968-015-0125-1
Normalization template	Further information: please see Papanastasiou et al, 2015, DOI:10.1186/s12968-015-0125-1
Noise and artifact removal	Further information: please see Papanastasiou et al, 2015, DOI:10.1186/s12968-015-0125-1
Volume censoring	Further information Papanastasiou et al, 2015, DOI:10.1186/s12968-015-0125-1

Statistical modeling & inference

Model type and settings	N/A
Effect(s) tested	N/A

Specify type of analysis: ☐ Whole brain ☒ ROI-based ☐ Both

Anatomical location(s)	Anatomical locations were identified based on characteristic skeletal anatomy, in consultation with experts in clinical research imaging.
Statistic type for inference (See Eklund et al. 2016)	N/A: Statistic type for inference and correction were not used as functional magnetic resonance imaging was not performed. For MRI, only anatomical imaging was performed.
Correction	N/A (see above: for MRI only anatomical imaging was performed)

Models & analysis

n/a	Involved in the study
☒	☐ Functional and/or effective connectivity
☒	☐ Graph analysis
☒	☐ Multivariate modeling or predictive analysis
